# Design of Multifunctional Tunable Metasurface Assisted by Elastic Substrate

**DOI:** 10.3390/nano12142387

**Published:** 2022-07-13

**Authors:** Jing Li, Hongjie Fan, Han Ye, Tiesheng Wu, Yuhang Sun, Xueyu Wang, Yumin Liu

**Affiliations:** 1 State Key Laboratory of Information Photonics and Optical Communications, Beijing University of Posts and Telecommunications, Beijing 100876, China; Li_jing@bupt.edu.cn (J.L.); hjanevan@bupt.edu.cn (H.F.); syh_19@bupt.edu.cn (Y.S.); xueyuwang@bupt.edu.cn (X.W.); 2 College of Information and Communication Engineering, Guilin University of Electronic Technology, Guilin 541004, China; tieshengw@guet.edu.cn

**Keywords:** tunable metasurface, beam splitter, elastic substrate, multifunctionality

## Abstract

Metasurfaces with both multifunctionality and tunability hold great application potential in next-generation optical devices. In this paper, we propose a stretchable metasurface composed of arrays of identical dielectric rectangular resonators embedded in the polydimethylsiloxane (PDMS) substrate. It is shown that the metasurface possesses three functions at the operating wavelength of 532 nm. The switching of functions can be implemented by changing the period *Px* of the metasurface, induced by stretching the PDMS substrate along the *x*-direction. When the period *Px* is less than the operating wavelength of 532 nm, the behavior of metasurface can switch between transmissive window and reflective mirror. When the period *Px* of the metasurface varies from 532 nm to 700 nm, the metasurface act as a dynamic equal-power beam splitter with conversion efficiency higher than 90%, and the corresponding splitting angle can be adjusted from 90° to around 49.5°. Moreover, we achieve the switching of transmissive window/reflective mirror/split-ratio-variable splitter based on the metasurface consisting of arrays of identical L-shaped resonators embedded in the PDMS substrate.

## 1. Introduction

Metasurfaces are a new type of planar optical elements composed of resonator arrays with subwavelength thickness. Unlike the conventional optical components and metamaterials, which shape the wavefront of light by gradually accumulating phases along the optical path, metasurfaces enable light control through abrupt phase shifts introduced by ultrathin resonators [1,2,3,4,5]. By carefully designing the structure and arrangement of the resonators, arbitrarily precise control of incident light can be realized. In the past decade, thanks to the advantages of compactness, lightweight, and arbitrary control over the wavefront, metasurfaces have developed rapidly and have been designed for a variety of applications, such as flat lenses [6,7,8], wave-plates [9,10,11], holograms [12,13,14], and beam splitters [15,16,17]. However, in general, once the metasurface is fabricated, its function and optical response range will be fixed, which will limit the practical application of metasurfaces. Thus, it is highly desired to achieve metasurfaces with tunability and multifunctionality.

There are two main methods for realizing the tunability of metasurfaces. One of the methods is to combine static metasurfaces with active materials, for example, liquid crystals [18,19,20], phase change materials [21,22,23,24], and transparent conducting oxides [25,26,27,28]. The optical response of such metasurfaces can be manipulated by adjusting the permittivity of the active material via external stimuli such as thermal [21] and electrical [18,19,22,23,24,25,26,27]. Another way to achieve tunable metasurfaces is to directly change the configuration of the metasurfaces, which can be achieved by combining metasurfaces with flexible substrates, such as embedding resonator arrays in flexible substrates or fabricating resonator arrays on flexible substrates. By applying mechanical stretching to the flexible substrates to change the geometric parameters of the metasurfaces, in-fine, dynamic control of the optical response can be achieved [29,30,31,32,33,34,35,36,37]. Since the optical properties of such metasurfaces are sensitive to changes in their shape and size, they have potential applications in the shape or displacement detection of objects [38,39]. Flexible metasurfaces allow for dynamic tuning of optical responses without the use of bias voltages and nonlinear elements, offering the advantages of simple structural design and low cost. Although many novel functions have been realized based on flexible optical metasurfaces, most flexible metasurfaces are currently only applied to the control of transmission, reflection, absorption, or resonance frequency [29,30,31,32,33,34]. There are few studies on the modulation of light wavefront [35,36]. As for multifunctionality, the most straightforward approach is to merge multiple single-function metasurfaces [40,41]. The advantage of this method is that multiple functions can be easily integrated into the same device, and the design is relatively simple. Nevertheless, such multifunctional metasurfaces will be severely affected by functional crosstalk. Another method is to integrate many distinct functions into a single metasurface [42,43], which is more complex in design than the first approach. However, metasurfaces designed based on this method can better solve the problems of functional crosstalk and low efficiency.

In this paper, we propose and demonstrate multifunctional tunable metasurfaces based on elastic substrates. By applying mechanical stretching to the elastic substrates, the proposed metasurfaces exhibit a transmissive window/reflective mirror/dynamic beam splitter transition. The metasurfaces are designed to operate at the common laser wavelength of 532 nm, but this design method can be applied at any wavelength.

## 2. Materials and Methods

**Figure 1:**
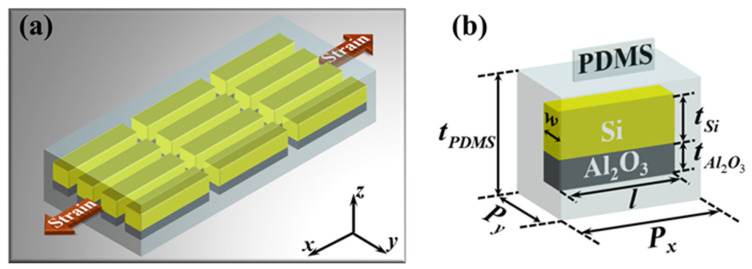
(**a**) Schematic of the proposed mechanically reconfigurable metasurface. (**b**) The three-dimensional structure diagram of a unit cell of the metasurface.

The schematic of the proposed metasurface is shown in Figure 1a, which is composed of an array of nanoblocks embedded in a PDMS substrate. PDMS, a flexible material with lower optical loss and remarkable elasticity, has been widely used in mechanically reconfigurable metasurfaces [29,30,31,32,33,34,35,36]. The unit cell of the metasurface is depicted in Figure 1b. It consists of a silicon (Si) rectangular cross-sectional nanoblock on a thin layer of aluminum oxide (Al_2_O_3_) embedded in PDMS substrate. The width *w* and length *l* of Si and Al_2_O_3_ is 80 nm and 370 nm, respectively. The thickness of Si and Al_2_O_3_ are fixed as *t*_Si_ = 110 nm and *t*_Al2O3_ = 50 nm. The thickness of PDMS substrate is denoted as *t_PDMS_*, the periods of the unit cell along the *x* and *y*-directions is *Px* and *Py*, respectively. Since the Young’s modulus of Si and Al_2_O_3_ are much larger than that of PDMS, Si and Al_2_O_3_ can be regarded as rigid materials during the stretching process. Thus, when the flexible substrate is mechanically stretched, the deformation of PDMS substrate will cause the physical distance between the nanoblocks, that is, the period of the metasurface, to change, while the optical constants and geometry of the nanoblocks remain unchanged. The numerical simulation is performed by three-dimensional finite-difference time-domain (FDTD) models. An *x*-polarized plane wave at a wavelength of 532 nm is normally incident from the bottom of PDMS substrate. The optical constants of Si and Al_2_O_3_ are taken from Ref [44] and the refractive index of 1.41 is used for PDMS. Perfectly matched layers in the *z*-direction and periodic boundary conditions along the *x*- and *y*-directions are applied in the simulation. In an unstrained state, the periods of the metasurface along the *x* and *y*-directions are set as *Px*_0_ = 450 nm and *Py*_0_ = 120 nm, the thickness of PDMS substrate is *t*_0_ = 3300 nm.

## 3. Results

**Figure 2:**
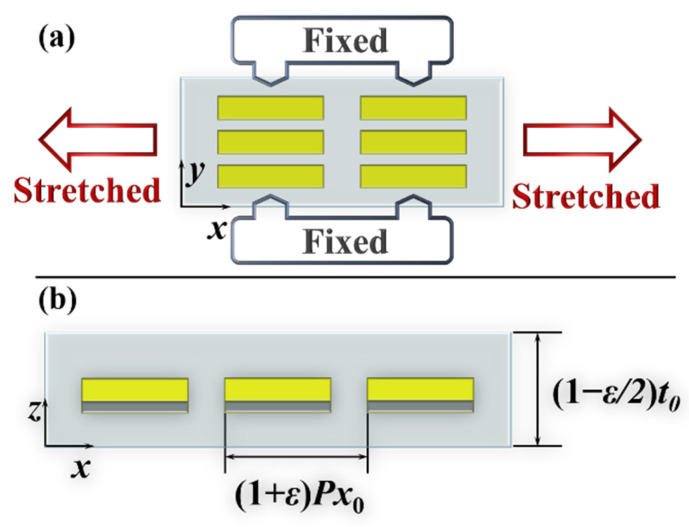
The schematic of the metasurface under uniaxially stretching with a strain ratio of *ε* in (**a**) *x–y* top view and (**b**) *x–z* side view.

In our work, we investigate the optical response of metasurfaces under two different stretching regimes, (1) fixing PDMS in the *y*-direction and stretching it along the *x*-direction; (2) stretching PDMS along the *x*-direction while unpinning it in the *y*-direction. Here, we first research the optical response of the metasurface under the first stretching method. This can be achieved by fixing PDMS with four clips of the fixture and applying uniaxially strain along the *x*-direction [30,32], as shown in Figure 2a (for details, see Appendix A). In this case, the period *Px* of the metasurface changes, while the period *Py* along the *y*-direction remains unchanged. Due to the uniformity of PDMS, we assume that the strain applied to the PDMS substrate can represent changes in the period of the unit cells. Thus, when a strain of *ε* is applied to the PDMS along the *x*-direction, *Px* can be expressed as *Px* = (1 + *ε*)*Px*_0_. The thickness of PDMS is reduced according to its Poisson’s ratio (*ν*), considered to be 0.5 here [29]. Hence, the thickness of PDMS is *t_PDMS_* = *t*_0_ − *vεt*_0_ = (1 − *ε*/2) *t*_0_, as shown in Figure 2b. In the case of normal incidence of *x*-polarized light, the operation of the metasurface will alter with the change of *Px*, and this phenomenon can be briefly numerically analyzed by the diffraction formula [45]:

(1) $$n_{t}\sin\theta_{t}-n_{i}\sin\theta_{i}=m\frac{\lambda_{0}}{Px}$$

where *n_t_* (*n_i_*) is the refractive index of the refracted (incident) medium, *θ_t_* (*θ_i_*) is the anomalous refraction (incident) angle, *λ*_0_ is the free-space wavelength of the light, *m* is the diffraction order of the metasurface, *Px* is the period of the unit cell along the *x*-direction. In our design, the incident and transmitted medium are both air (*n_t_* = *n_i_* = 1), *θ_i_* = 0. Importing these parameters into Equation (1), we can obtain the relationship between the splitting angle and *Px* as below:

(2) $$\sin\theta_{t}=m\frac{\lambda_{0}}{Px}$$

The total number of all diffraction orders can be calculated based on the ratio of the incident wavelength to the period according to Equation (2). When *Px* is smaller than wavelength *λ*, no diffraction takes place, that is, there is only zeroth order in transmission or reflection mode. The diffraction phenomenon occurs when *Px* is larger than *λ*. When *λ*_0_/*P* is greater than 0.5 but less than 1, the metasurface will have three diffraction orders: −1, 0, and +1 [46]. Hence, for the operating wavelength of 532 nm, only −1, 0, and +1 diffraction orders exist when the period of the metasurface is in the range from 532 nm to 1064 nm. Accordingly, we analyze the two cases where *Px* is less than the wavelength *λ* and *Px* is greater than the wavelength *λ*.

**Figure 3:**
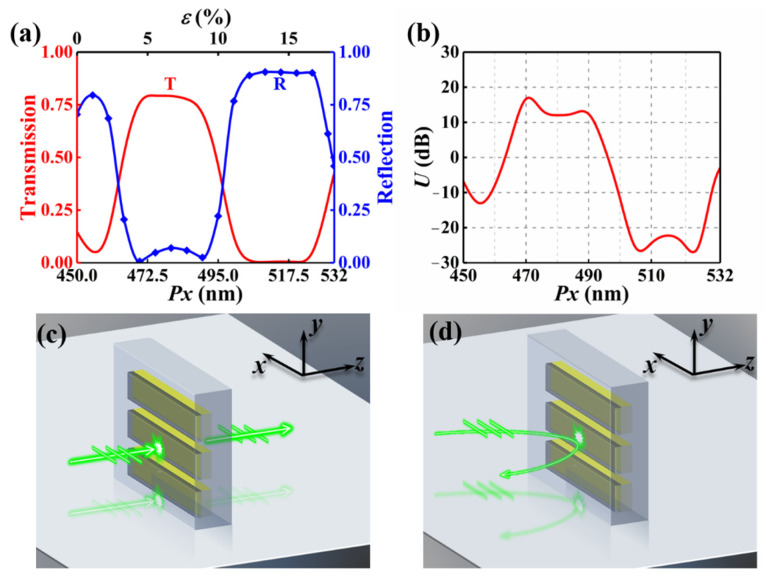
The optical response of the metasurface when *Px* is smaller than the wavelength *λ*. (**a**) Transmission and reflection of the metasurface versus period. (**b**) *U* as a function of *Px*. The working mechanism of the design operating as (**c**) transmissive window and (**d**) reflective mirror.

First, we explore the optical response of the metasurface when *Px* is smaller than the wavelength *λ*. The transmission (*T*) and reflection (*R*) of metasurface when *Px* varies from 450 nm to 532 nm at the wavelength of 532 nm are depicted in Figure 3a. It can be seen that the transmission peak and reflection peak alternate with the change of *Px*. We use an index *U*, defined as *U*(dB) = 10log10(*T*/*R*), to evaluate the unidirectionality. Positive (negative) *U* means the transmission is higher (lower) than reflection. We define the metasurface as a transmissive window when *U* is greater than 10 dB, and as a reflective mirror when *U* is less than −10 dB. Figure 3b shows *U* as a function of *Px*. The behavior of metasurface alters from transmissive window (Behavior-Ι) to reflective mirror (Behavior-ΙΙ). For Behavior-Ι, the corresponding period ranges from 467 nm to 492 nm. The working schematic is shown in Figure 3c, the incident light is almost completely transmitted through the metasurface, while the reflected light is almost perfectly suppressed. For Behavior-ΙΙ, the working mechanism is shown in Figure 3d, the incident light is almost completely reflected by the metasurface. The corresponding period *Px* ranges from 500 nm to 530 nm, particularly, *U* is less than −20 dB within the period *Px* region from 502 nm to 526 nm. It should be pointed out that *U* is less than −10 dB when *Px* is changed from 452 nm to 458 nm, but the region is too narrow, so it is not considered in our work.

**Figure 4:**
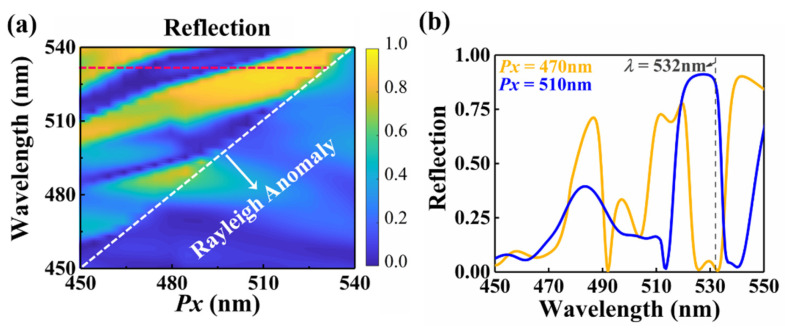
(**a**) Reflection as a function of the period *Px* and wavelength. (**b**) Reflection as a function of wavelength for the metasurface with *Px* = 470 nm and *Px* = 510 nm.

We also investigate the physical mechanism of this phenomenon in which the behavior of the metasurface switches from a transmissive window to a reflective mirror. Figure 4a shows the reflection spectrum as a function of period *Px* and wavelength. The white dashed line represents the ±1st order Rayleigh anomaly [47], and the spectral position can be determined by the simplified formula *λ* = *Px*. As depicted in Figure 4b, for metasurfaces with a certain period *Px*, when *Px* < *λ*, there are multiple perfect transmission peaks and perfect reflection peaks, which provides the possibility for the behavior switching between the transmissive window and the mirror of our proposed metasurface. These reflection peaks and transmission peaks are mainly caused by the combinations of multipolar interferences and lattice coupling. The reflection peaks mainly rely on lattice coupling. In contrast, the transmission peaks are weakly affected by lattice coupling, they mainly depend on multipolar interference [48,49].

**Figure 5:**
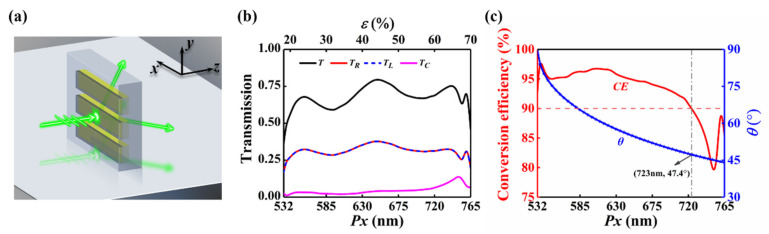
The optical response of the metasurface when *Px* is larger than the wavelength *λ*. (**a**) The working mechanism of the design operating as equal-power splitter. (**b**) Total transmission and intensity of the three emergent beams of the metasurface as a function of period. (**c**) Conversion efficiency and splitting angle as a function of period.

When the period *Px* is greater than the operating wavelength of 532 nm, the working mode of the metasurface changes. According to Equation (2), the diffraction phenomenon will occur when the period *Px* is greater than the wavelength. In our previous study [43], it has been confirmed that such uniform metasurfaces possess efficient and broadband beam-splitting performance as shown in Figure 5a. After passing through the metasurface, the incident light is divided into three parts that propagate along the left side (negative *x*-axis direction) of the normal, the right side (positive *x*-axis direction) of the normal, and the normal direction. For beam splitters, conversion efficiency (*CE*), defined as *CE* = (*T_L_* + *T_R_*)/*T* × 100%, is one of the key factors to measure the beam splitting performance. We define that when the conversion efficiency is higher than 90%, the metasurface exhibits efficient beam splitting performance. Figure 5b depicts the transmission intensity of the three parts of the outgoing light and the total transmission when *Px* varies from 532 nm to 765 nm (*ε* = 70%). The elastic limit of PDMS is around 200% [50], so a period *Px* of 765 nm is achievable by stretching the PDMS substrate. It can be seen that *T_R_* and *T_L_* are always the same at any wavelength. According to Equation (2), the angle between the emergent light beams propagating on the left and right sides of the normal and the normal direction is the same, which we define as the splitting angle *θ*. Figure 5c depicts the conversion efficiency *CE* and splitting angle *θ* of the metasurface when the period *Px* increases from 532 nm to 765 nm. The conversion efficiency remains higher than 90% within the period *Px* region from 532 nm to 723 nm, where the splitting angle varies from 90° to 47.4°. Thus, the device operates as an efficient dynamic equal-power splitter (Behavior-ΙΙΙ) when the period *Px* varies from 532 nm to 723 nm, the corresponding working mechanism is depicted in Figure 5a.

**Figure 6:**
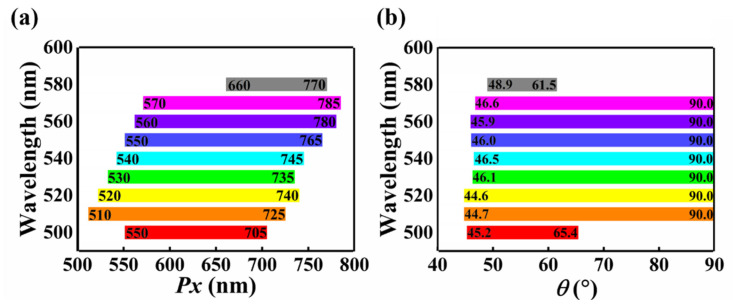
(**a**) The range of period *Px* of metasurfaces that can be used as efficient dynamic beam splitters for specific wavelengths. (**b**) The corresponding splitting angle of metasurfaces with different period *Px* for specific wavelengths.

The above analysis indicates that such metasurface exhibits a transmissive window/reflective mirror/efficient dynamic beam splitter transition at the operating wavelength of 532 nm when the period *Px* increases from 467 nm to 723 nm. We also explore the wavelength dependence of this dynamic modulation behavior. The results demonstrate that the metasurface can only achieve this switching of behavior modes with mechanical stretching at wavelengths around 532 nm (525 nm to 538 nm). Although the proposed metasurface cannot achieve the switching of multiple operating modes in a broadband, it can be used as a dynamic beam splitter (*Px* > *λ*) in a bandwidth of 80 nm, as shown in Figure 6a. Figure 6a shows the corresponding *Px* region in which the design can be used as an effective beam splitter at certain wavelengths. Figure 6b depicts the variation range of the splitting angle *θ* when the metasurface is used as an efficient dynamic beam splitter at some specific wavelengths. Although we only present the dynamic beam splitting performance of the proposed metasurface at certain wavelengths in Figure 6a,b, in fact, in the wavelength range from 500 nm to 580 nm, the metasurface can be used as a mechanically reconfigurable dynamic beam splitter, and the splitting angle range covers from 44.6° to 90°.

**Figure 7:**
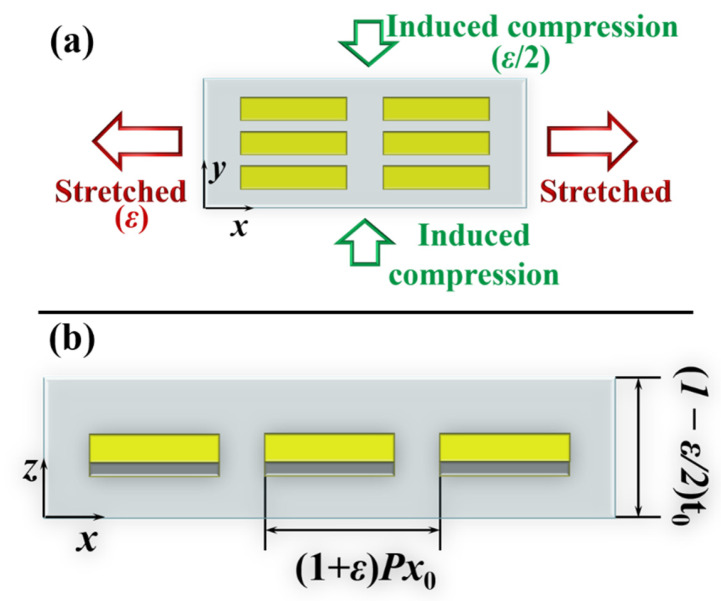
The schematic of the metasurface under uniaxially stretching with a strain ratio of *ε* in (**a**) *x*–*y* top view and (**b**) *x*–*z* side view. Both ends of PDMS in the *y*-direction are not fixed.

Here, we research the dynamic manipulation of the optical response of the metasurface under the second stretching method, as shown in Figure 7a. Since PDMS is an isotropic material with a Poisson’s ratio of *ν* = 0.5, the stretching with a strain ratio of *ε* along the *x*-direction shall induce half compressions along the *y*-direction and *z*-direction. Hence, *Px*, *Py* and *t_PDMS_* can be expressed as *Px* = (1 + *ε*)*Px*_0_, *Py* = *Py*_0_ − *νεPy*_0_ = (1 − *ε*/2)*Py*_0_, and *t_PDMS_* = *t*_0_ − *νεt*_0_ = (1 − *ε*/2)*t*_0_, as shown in Figure 7. For this case, *Py*_0_ is set as 130 nm in the unstrained state, while other parameters remain unchanged. 

**Figure 8:**
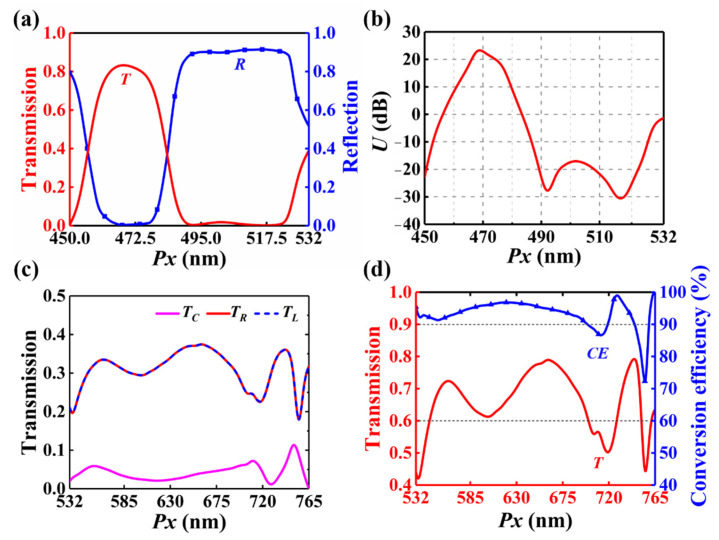
(**a**) Transmission and reflection of the metasurface and (**b**) *U* versus period when *Px* is smaller than the wavelength *λ*. (**c**) Intensity of the three emergent beams as a function of period. (**d**) Conversion efficiency and total transmission when *Px* is greater than the wavelength *λ*.

Figure 8a shows the transmission and reflection of this metasurface when the period *Px* is changed from 450 nm to 532 nm. In this case, the behavior of the metasurface can also alter from a transmissive window (Behavior-Ι) to a reflective mirror (Behavior-ΙΙ). Figure 8b depicts *U* as a function of *Px*. For Behavior-Ι, the corresponding period ranges from 462 nm to 480 nm. For Behavior-ΙΙ, the corresponding period *Px* region covers from 488 nm to 527 nm. Figure 8c depicts the transmission of the three parts of the emergent light when *Px* varies from 532 nm to 765 nm, indicating that this design operates as an equal-power beam splitter. Transmission and conversion efficiency as a function of *Px* is shown in Figure 8d. Conversion efficiency remains higher than 90% within the period region from 532 nm to 700 nm, where the splitting angle varies from 90° to 49.5°.

## 4. Discussion

**Figure 9:**
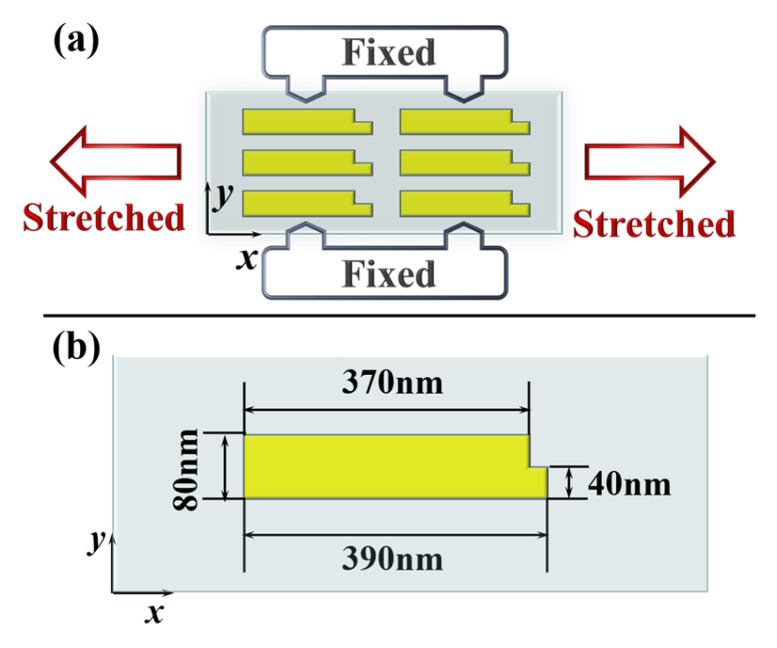
The schematic of (**a**) the metasurface composed of an array of L-shaped antennas embedded in a PDMS substrate in *x–y* top view and (**b**) unit cell in *x–y* view.

The above analysis shows that our proposed metasurface composed of the array of identical nanoblocks embedded in the PDMS substrate can achieve transmissive window/reflective mirror/dynamic equal-power beam splitter operating mode switching under mechanical stretching. In some coherent systems, split-ratio-variable splitter is desired to control the interference of two beams. Here, we propose a metasurface consisting of an array of L-shaped antennas embedded in a PDMS substrate. The schematic of the metasurface under uniaxially stretching with a strain ratio of *ε* in *x–y* top view is shown in Figure 9a. The geometric asymmetry of the L-shaped antennas enables unequal power distribution of the outgoing beams propagating on the left and right sides along the normal direction. Figure 9b shows the geometric parameters of L-shaped antenna in *x–y* view. Other geometric parameters remain unchanged.

**Figure 10:**
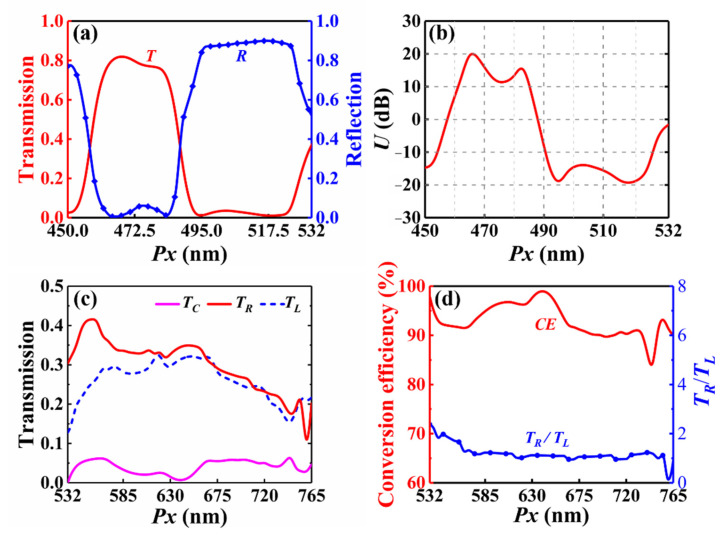
(**a**) Transmission and reflection and (**b**) *U* of the metasurface versus period when *Px* is smaller than the wavelength λ. (**c**) Intensity of the three emergent beams of the metasurface as a function of period. (**d**) Conversion efficiency and the split ratio between *T_R_* and *T_L_* as a function of period.

Figure 10a shows the transmission and reflection of metasurface when the period *Px* is changed from 450 nm to 532 nm. In this case, the behavior of the metasurface can also alter from a transmissive window (Behavior-Ι) to a reflective mirror (Behavior-ΙΙ). Figure 10b depicts *U* as a function of *Px*. For Behavior-Ι, the corresponding period ranges from 462 nm to 485 nm. Reflection is more than 10 times higher than transmission when the period *Px* changes from 491 nm to 525 nm (Behavior-ΙΙ). Figure 10c depicts the transmission of the three parts of the emergent light when *Px* varies from 532 nm to 765 nm, indicating that the design operates as a split-ratio-variable splitter. Conversion efficiency and the split ratio between *T*_+1_ and *T*_−1_ as a function of *Px* are shown in Figure 10d. Conversion efficiency remains higher than 90% within the period region from 532 nm to 735 nm, and the corresponding splitting angle varies from 90° to 46.4°. As the *Px* increases from 532 nm to 615 nm, the split ratio is effectively decreased from 2.38 to 1.03. For larger *Px*, the tunability of the split ratio is reduced, and the split ratio stabilizes around 1. Thus, in this case, the metasurface can exhibit a transmissive window/reflective mirror/split-ratio-variable splitter transition under external mechanical stretching.

## 5. Conclusions

In summary, we present a heuristic scheme for designing trifunctional tunable metasurfaces based on PDMS substrate. The deformation of a substrate under stretching induces a change in the configuration of the metasurface, thus affecting the resonance coupling between unit cells, and, eventually, achieving the dynamic manipulating of the optical response. Two different stretching methods are investigated: (1) fixing PDMS in the *y*-direction and stretching PDMS along the *x*-direction; (2) uniaxial stretching PDMS along the *x*-direction. The results show that our design consistently exhibits a transmissive window/reflective mirror/dynamic equal-power splitter transition under two different stretching regimes when applying mechanical strain ranging from 0% to 70% on the PDMS substrate. This conversion mechanism is also investigated. Moreover, by applying asymmetric resonators in the metasurface, we achieve the switching of transmissive window/reflective mirror/split-ratio-variable splitter. We hope that the proposed metasurface can be used in next-generation optical devices.

## Data Availability

Data sharing is not applicable to this article.

## Appendix A. Experimental Feasibility

Here, with support from some of the reported literature [29,51], we provide the practicable experimental processes and optical measurements. For the fabrication, first, on the silicon wafer, a germanium sacrificial layer is deposited by electron-beam evaporation, and then the silicon layer is deposited by plasma-enhanced chemical vapor deposition on the germanium layer. The metasurface pattern can be written using electron beam lithography (EBL). Second, the aluminum oxide layer is deposited on the patterned silicon by e-beam evaporation, the silicon pattern is then dry etched adopting aluminum oxide as the hard mask. Then, the mixture of PDMS polymer diluted with toluene is first spin-coated on the sample to fill the nanoblock gaps and form a thin PDMS layer, and then the undiluted PDMS is spin-coated again on the sample to form a thicker PDMS film. Finally, the nanoblocks embedded in PDMS are released by dissolving the germanium layer. A layer of diluent-free PDMS is then spin-coated on the other side of the sample to fully encapsulate the nanoblocks in PDMS. The strain is applied on the PDMS substrate along the *x*-direction to investigate the mechanically tunable response of the metasurface. The sample is held by four clamps with each of them mounted on an identical linear translation stage. As the two linear stages in the *x*-direction are translated by the same amount, the metasurface located at the center of the PDMS film is stretched. The sample is illuminated by a normally incident collimated beam that is emitted by a 532 nm laser via a linear polarizer. Since the footprints of the metasurfaces fabricated in the experiments are usually small, an objective is required to fully focus the incident light on the metasurface area. A detector appended to a spectrometer is used to capture the transmission and reflection. Real-space and k-space (diffraction orders) images of the sample can be captured by the CCD, respectively.
